# *Achatina fulica *infected by *Angiotrongylus cantonensis* in Napo, Ecuador

**DOI:** 10.17843/rpmesp.2022.391.10148

**Published:** 2022-03-31

**Authors:** Luis Solórzano Álava, Hilda Hernández Álvarez, Cesar Bedoya Pilozo, Misladys Rodríguez, Lázara Rojas Rivero

**Affiliations:** 1 Instituto Nacional de Investigación en Salud Pública (INSPI), Guayaquil, Ecuador. Instituto Nacional de Investigación en Salud Pública (INSPI) Guayaquil Ecuador; 2 Instituto Pedro Kouri, La Habana, Cuba. Instituto de Medicina Tropical Pedro Kourí Instituto Pedro Kouri La Habana Cuba

*To the editor*. *Angiostrongylus cantonensis* is a zoonotic parasite recognized as one of the main pathogens associated with meningitis and/or eosinophilic meningoencephalitis ^1^. In 2008, the first outbreak of natural transmission of *A. cantonensis* was described in Ecuador. It is currently a neglected parasitosis with evident underreporting of cases ^2^, even though it is one of the four zoonotic events notifiable to the Ministry of Public Health (MSP). In 2019, the surveillance system (SIVE-ALERT) of the MSP reported 85 cases of leptospirosis, 27 of brucellosis, and no cases of rabies and plague during the previous ten years. As for eosinophilic meningitis, the last case was officially reported in 2017 ^3^.

The life cycle of this parasite requires definitive and intermediate hosts. It is known that rats are the definitive hosts of *A. cantonensis*, after ingestion of the L3 larvae, which migrate to the central nervous system (CNS) where they mature into L4 and L5 larvae, developing into adult worms. When they reach sexual maturity, they lay their eggs in the pulmonary arteries; these develop into L1 larvae that migrate to the trachea where they are swallowed, passing into the gastrointestinal tract and excreted in the feces. Feces with larvae are ingested by snails that act as intermediate hosts, and progress to the L3 larval stage (infective larva for the definitive and accidental host). Humans are not the definitive host of the parasite, but become an accidental host by ingesting intermediate hosts or carriers containing infected larvae. In humans, the parasite does not complete its life cycle, but remains in the CNS, causing eosinophilic meningitis, or may settle in the eye chamber, causing ocular angiostrongyliasis ^4^.

In previous studies on snails in several regions of the country, the province of Napo shows high infection rates of 30.0% (unpublished data from the Instituto de Investigación en Salud Pública [INSPI]) and 27.16% ^2^. Therefore, the aim of this research, which included a larger number of intermediate hosts and a larger geographical extension, was to establish the infection rate and the average intensity of infection by *A. cantonensis* in the *Achatina fulica* snail in three cantons of the Napo province (Archidona, Tena and Arosemena Tola).

Between June and September 2019, specimens of the *A. fulica* snail were collected in the Napo province, which has an area of 13,271 km^2^, an altitude of 500 MSL, rainfall of 5000 mm per year, a temperature of 25 °C and humidity of 90 to 100%. The manual capture of these intermediate hosts was carried out using the method of capture per unit effort in 15 min ^2^. The snails were transported to the INSPI Laboratory, where they were examined for the presence of L3 larvae of *A. cantonensis* using the Lobato Paraense method for the extraction of soft organs ^5^. The paleal membrane was separated and placed in digestion solution for 6 hrs ^6^. L3 larvae were identified using a Motic SMZ-168 ™ stereomicroscope and an Olympus CX31 ^TM^ microscope. The percentage of infection and the average intensity of infection by *A. cantonensis* in intermediate hosts were determined as follows:


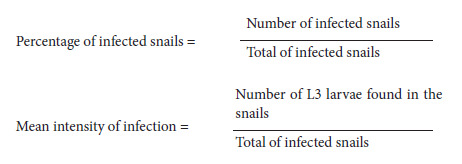


A total of 1476 specimens were collected, the percentage of snails infected with *A. cantonensis* was 46.5% (687/1476). The number of larvae found in the 687 infected *A. fulica* was 2236, i.e., the average intensity of infection was 3.3 L3 larvae per infected snail (Table 1).

**Table 1:** Percentage of intermediate hosts infected with *A. cantonensis* in three cantons of Napo (Tena, Carlos Julio Arosemena, Archidona) Ecuador.

Studied Cantons	Location	Number of collected specimens	Number of infected snails	Percentage of infection in the snails	Number of L3 larvae	Mean intensity of the infection
Archidona	Archidona	100	54	54.0	272	5.0
Tena	Ahuano	120	59	49.2	212	3.6
	Pano	100	30	30.0	90	3.0
	Puerto Napo	100	66	66.0	125	1.9
	Misahuallí	100	60	60.0	168	2.8
	Talag	135	24	17.8	110	4.6
	Casa del Diabético	100	45	45.0	60	1.3
	Paushiyacu	100	42	42.0	197	4.7
	Urbano Tena	100	23	23.0	70	3.0
	Satelital Tena	100	61	61.0	183	3.0
	Muyuna	100	55	55.0	176	3.2
	Surroundings of the Hospital José M. Velas co Ibarra	100	54	54.0	80	1.5
	Shandia	121	44	36.4	150	3.4
Total Arosemena	Total Arosemena	100	70	70.0	70.0	4.9
	Total	1476	687	46.5	2236	3.3

L3: Infective larva for the definitive and accidental host

The study included only the species *A. fulica* because it is the most prolific intermediate host species, has the highest number of viable eggs, is terrestrial and was found in greater numbers in the studied locations, unlike other intermediate host species that are found in freshwater.

The results reveal a wide distribution of *A. fulica* infected with *A. cantonensis* and a higher percentage of infection than what has being reported by previous studies. These results have implications for the zoonotic transmission of the parasite, as human infection is mediated by infected intermediate hosts; ingestion is the main route of transmission, and shows that a considerable number of people are at risk of being infected, either through existing culinary traditions or through contamination of food with the infecting larvae. Strategies to minimize human infection should include community-targeted educational interventions, snail control to reduce the likelihood of ingestion, proper cooking of intermediate hosts, and education on the preparation of food to prevent the occurrence of sporadic cases and outbreaks of the disease.
