# Supplementary material for: Achatina fulica infected by Angiotrongylus cantonensis in Napo, Ecuador
Source: Rev Peru Med Exp Salud Publica. 2022 Mar 31;39(1):122–3. doi: 10.17843/rpmesp.2022.391.10148 (PMC11397689; doi:10.17843/rpmesp.2022.391.10148)
Supplement: Translation

# Translation: *Achatina fulica* infectado por *Angiostrongylus cantonensis *en Napo, Ecuador

**Authors:** Luis Solórzano Álava, Hilda Hernández Álvarez, Cesar Bedoya Pilozo, Misladys Rodríguez, Lázara Rojas Rivero

**Affiliations:** 1 Instituto Nacional de Investigación en Salud Pública (INSPI), Guayaquil, Ecuador.; 2 Instituto Pedro Kouri, La Habana, Cuba.

Sr. Editor. *Angiostrongylus cantonensis *es un parásito zoonótico reconocido como unos de los principales patógenos asociados con meningitis y/o meningoencefalitis eosinofílica ^1^. En el 2008 se describe el primer foco de transmisión natural de *A. cantonensis* en Ecuador. Actualmente es una parasitosis desatendida con evidente subregistro de casos ^2^, a pesar de que es uno de los cuatro eventos zoonóticos de notificación obligatoria al Ministerio de Salud Pública (MSP). Según el sistema de vigilancia (SIVE-ALERTA) del MSP, en el 2019 se reportaron 85 casos de leptospirosis, 27 de brucelosis, y ningún caso de rabia y peste en los últimos diez años. En cuanto a la meningitis eosinofílica, en el 2017 se reportó oficialmente el último caso ^3^_._

El ciclo de vida de este parásito requiere de hospederos definitivos e intermediarios. Se conoce que las ratas son los hospederos definitivos de *A. cantonensis*, luego de la ingestión de larvas L3, estas migran al sistema nervioso central (SNC) donde maduran a un estado de larvas L4 y L5, desarrollándose en gusanos adultos. Cuando alcanzan la madurez sexual ponen sus huevos en las arterias pulmonares; estos se convierten en larvas L1 que migran a la tráquea donde son deglutidas, pasando al tracto gastrointestinal y excretadas por las heces. Las heces con larvas son ingeridas por caracoles que actúan como hospederos intermediarios, y progresan a la fase larvaria L3 (larva infecciosa para el hospedero definitivo y accidental). Los humanos no son el hospedero definitivo del parásito, pero se convierten en accidentales al ingerir hospederos intermedios o transportadores que contienen larvas infectadas. En el hombre, el parásito no completa el ciclo de vida, sino que permanece en el SNC lo que origina una meningitis eosinofílica o puede instalarse en la cámara de ojo produciendo una angiostrongiliasis ocular ^4^. 

En estudios previos de caracoles en varias regiones del país, la provincia de Napo muestra tasas altas de infección, 30,0% (datos no publicados del Instituto de Investigación en Salud Pública [INSPI]) y 27,16% ^2^. Teniendo en cuenta lo anterior, se realizó la presente investigación que incluyó un número mayor de hospederos intermediarios y una extensión geográfica mayor, con el fin de establecer el porcentaje de infección y la intensidad promedio de la infección por *A. cantonensis* en el caracol *Achatina fulica*, de tres cantones de la provincia Napo (Archidona, Tena y Arosemena Tola). 

Entre junio y septiembre del 2019, se recolectaron especímenes del caracol *A. fulica* en la provincia Napo la cual tiene una superficie de 13 271 km^2^, una altitud de 500 m s.n.m, con precipitaciones de 5000 mm al año, una temperatura de 25 ºC y humedad de 90 a 100%. La captura manual de estos hospederos intermediarios se realizó mediante el método de captura por unidad de esfuerzo en 15 min ^2^. Los caracoles fueron transportados al Laboratorio del INSPI, donde fueron examinados para evidenciar la presencia de larvas L3 de *A. cantonensis* utilizando el método de Lobato Paraense para la extracción de los órganos blandos ^5^. La membrana paleal se separó y se colocó en solución de digestión durante 6 h ^6^. Las larvas L3 se identificaron, utilizando un estereomicroscopio Motic SMZ-168 ™ y un microscopio Olympus CX31 ^TM^. Se determinó el porcentaje de infección y la intensidad promedio de la infección por *A. cantonensis *en hospederos intermediarios de la siguiente manera:



Se recolectaron 1476 especímenes, el porcentaje de caracoles infectados con *A. cantonensis* fue de 46,5% (687/1476). El número de larvas encontradas en los 687 *A. fulica* infectados fue de 2236, es decir, la intensidad promedio de infección fue de 3,3 larvas L3 por caracol infectado (Tabla 1).

**Tabla 1:** Porcentaje de infección de *A. cantonensis* en hospederos intermediarios de tres cantones de Napo (Tena, Carlos Julio Arosemena, Archidona) Ecuador.

Cantones estudiados	Localidades	Número de ejemplares recolectados	Número de caracoles infectados	Porcentaje de infección en los caracoles	Número de larvas L3	Intensidad promedio de la infección
Archidona	Archidona	100	54	54,0	272	5,0
Tena	Ahuano	120	59	49,2	212	3,6
	Pano	100	30	30,0	90	3,0
	Puerto Napo	100	66	66,0	125	1,9
	Misahuallí	100	60	60,0	168	2,8
	Talag	135	24	17,8	110	4,6
	Casa del Diabético	100	45	45,0	60	1,3
	Paushiyacu	100	42	42,0	197	4,7
	Urbano Tena	100	23	23,0	70	3,0
	Satelital Tena	100	61	61,0	183	3,0
	Muyuna	100	55	55,0	176	3,2
	Alrededores del Hospital José M. Velasco Ibarra	100	54	54,0	80	1,5
	Shandia	121	44	36,4	150	3,4
Arosemena Total	Arosemena Tola	100	70	70,0	343	4,9
	Total	1476	687	46,5	2236	3,3

L3: larva infecciosa para el hospedero definitivo y accidental.

El estudio incluyó solo la especie* A. fulica *debido a que es la especie de hospedero intermediario más prolífico, con mayor número de huevos viables, además al ser terrestre, se encontró en mayor número en las localidades estudiadas, a diferencia de otras especies de hospederos intermediaros que son dulceacuícolas.

Los resultados revelan una amplia distribución de *A. fulica* infectado con *A. cantonensis* y un porcentaje de infección mayor que en estudios previos. Estos resultados tienen implicaciones para la transmisión zoonótica del parásito, ya que la infección humana se da en función de hospederos intermediarios infectados, cuya ingestión es la principal vía de transmisión, lo que indica que un número considerable de personas está en riesgo de adquirir la infección, ya sea por las tradiciones culinarias existentes o por la contaminación de alimentos con las larvas infectantes. Las estrategias para minimizar la infección en humanos deben incluir intervenciones educativas dirigidas a la comunidad, control de caracoles para reducir las posibilidades de ingestión, cocción de hospederos intermediarios y educación sobre la preparación de alimentos para prevenir la aparición de casos esporádicos y de brotes de la enfermedad.
